# Full-length transcriptome sequencing of *Arabidopsis* plants provided new insights into the autophagic regulation of photosynthesis

**DOI:** 10.1038/s41598-024-65555-7

**Published:** 2024-06-25

**Authors:** Song Wang, Yunfeng Shi, Yanhui Zhou, Weiming Hu, Fen Liu

**Affiliations:** 1 Lushan Botanical Garden, Jiangxi Province and Chinese Academy of Sciences, Jiujiang, 332900 Jiangxi China; 2 College of Life Science, Nanchang University, Nanchang, 330031 Jiangxi China

**Keywords:** Full-length transcriptome, Autophagy, Photosynthesis, qRT-PCR, *Arabidopsis thaliana*, Computational biology and bioinformatics, Gene regulatory networks, Plant sciences, Photosynthesis, Photosystem II

## Abstract

Autophagy is a highly conserved eukaryotic pathway and plays a crucial role in cell survival under stress conditions. Here, we applied a full-length transcriptome approach to study an *Arabidopsis* autophagy mutant (*atg5-1*) subjected to nitrogen-starvation, using Oxford Nanopore Technologies. A total of 39,033 transcripts were identified, including 11,356 new transcripts. In addition, alternative splicing (AS) events and lncRNAs were also detected between Col-0 (WT) and *atg5-1*. Differentially expressed transcript enrichment showed that autophagy upregulates the expression of many stress-responsive genes and inhibits the transcription of photosynthesis-associated genes. The qRT-PCR results showed that the expression patterns of photosynthesis-related genes in the *atg5-1* differed under the conditions of nitrogen starvation and carbon starvation. Under nitrogen starvation treatment, many genes related to photosynthesis also exhibited AS. Chlorophyll fluorescence images revealed that the Fv/Fm and ΦPSII of old *atg5-1* leaves were significantly reduced after nitrogen starvation treatment, but the *Y*(*NPQ*) indices were significantly increased compared to those of the WT plants. The results of qRT-PCR suggest that autophagy appears to be involved in the degradation of genes related to photodamage repair in PSII. Taken together, the full-length transcriptiome sequencing provide new insights into how new transcripts, lncRNAs and alternative splicing (AS) are involved in plant autophagy through full-length transcriptome sequencing and suggest a new potential link between autophagy and photosynthesis.

## Introduction

In response to stress, plants use a variety of adaptive responses to maintain an adequate supply of nutrients for growth, development, reproduction, and protection. One important pathway involves the autophagic turnover of intracellular substances, which is essential for the proper processing of unnecessary or dysfunctional substances in the cells and the subsequent reuse of the nutrient components^1,2^. To date, three different types of autophagy namely, microautophagy, macroautophagy, and mega-autophagy, have been identified in plants^3,4^. The best-studied type of autophagy in plants is macroautophagy, in which autophagosomes are formed and then fuse with vacuoles to break down cargos^5,6^. Over the past decade, more than 40 ATG (autophagy-related gene) proteins have been identified in the canonical macroautophagy pathway have been identified in *Arabidopsis thaliana*^1,7^. These proteins can be classified into four groups: the ATG1/ATG13 kinase complex, the phosphatidylinositol 3-kinase (PI3K) complex, the ATG9 complex, and the ATG8 and ATG12 ubiquitin-like conjugation systems^6,8–10^. To date, many regulatory factors, including TOR, SnRK1, FREE1, and SH3P2, have been shown to be involved in the regulation of autophagy in plants^11–14^. In addition, multiomic methods, including transcriptomic, metabolomic, and proteomic methods, have been used to dissect autophagy regulatory networks^15–18^.

Research has increasingly shown that autophagy acts as a protective strategy for plants to cope with a variety of stresses. Under stress, the expression of many *ATG* genes is rapidly upregulated simultaneously to promote autophagy, recycle nutrients, and remove harmful debris to maintain cell homeostasis^19,20^. Conversely, autophagy mutants (e.g., *atg5-1*) become chlorotic or exhibit premature senescence under stress^17,21,22^, further indicating the important role of autophagy in plant resistance to abiotic stress. However, until now, only a few transcriptional regulators involved in these processes have been identified in *Arabidopsis*^8^.

Transcriptional regulation is an important step in regulating eukaryotic gene expression. In Arabidopsis, transcriptional changes in *ATG* genes are frequently observed during plant development and adaptation to environmental changes^23–28^. Using conventional transcriptome technology, Masclaux-Daubresse et al. revealed connections between autophagy and salicylic acid biosynthesis and response, cytokinin perception, oxidative stress, and plant defense^15^. In another transcriptomic study, Minina et al. reported that genes associated with necrotrophic pathogens and oxidative stress were abundant in *ATG5*- or *ATG7*-overexpressing plants^17^. However, the exploration of the transcriptional regulation of autophagy genes by third-generation sequencing has not been explored. Compared with traditional second-generation transcriptome sequencing, third-generation full-length sequencing based on the Oxford Nanopore Technologies (ONT) platform can more accurately and variably splice alternative transcripts (AS), long noncoding RNAs (lncRNAs) and their target genes^29^. A recent study showed that this technique provides better-quality raw data and more accurate estimates of transcription levels than the PacBio technique^29^. The ONT platform has been widely applied for whole-genome sequencing, but has rarely been used for full-length transcriptome sequencing^30^.

In this study, we generated 12 full-length transcriptomes of wild-type *Arabidopsis thaliana* and the autophagy mutant *atg5-1* under low and high nitrogen levels using the ONT platform. Based on the above data, we first analyzed the differentially expressed transcripts (DETs) between the different groups. Compared with *atg5-1*, the WT had a greater number of stress-related DETs whose expression increased expression after nitrogen starvation, while the expression of photosynthesis-related DETs decreased. The indices obtained from the chlorophyll fluorescence images also further proved the difference between the WT and *atg5-1* in terms of their photosynthetic capacity. The above sequencing results were further verified by determination of photosynthetic indicators.

Although several articles on the autophagy-related transcriptome have been published, no studies on dynamic AS events and lncRNA-regulated target genes have been published. In this study, we examined AS events and identified lncRNAs under nitrogen starvation conditions. AS is a common way to increase protein diversity after transcription^31^. Several studies have provided an overwhelming amount of unassembled data for on AS, as they produce diverse and high-quality transcripts of different lengths, such as AS events that occur in response to drought stress^32^ and those that occur during development^33,34^. AS enriches the diversity of the transcriptome and proteome and offers more flexibility in transcriptional regulation^35^. In this study, we found that many transcripts related to photosynthesis exhibited AS. LncRNAs are involved in gene transcription and posttranscriptional regulation in eukaryotes^36–39^. LncRNAs can regulate target genes by being transcribed, sequestering microRNAs and proteins, or acting as guide RNAs to recruit proteins^40,41^. LncRNAs have been closely implicated in metabolism, flowering, fertility, and the biotic and abiotic stress response in plants^42–44^. We also detected differences in the expression of several lncRNAs after nitrogen starvation between the WT and *atg5-1* plants. Taken together, our results provide a basis for further understanding the mechanism of autophagy regulation and the relationship between autophagy and photosynthesis.

## Results

### Analysis of ONT sequencing datasets

To detetmine the defined transcriptional regulation of autophagy in Arabidopsis after nitrogen starvation, Col-0 and *atg5-1* mutants were subjected to full-length ONT transcriptome sequencing. After sequencing, the original fastq data were filtered for short fragments and low-quality reads, resulting in clean data. A total of 62 GB of clean data were produced with 4,522,054 to 5,597,930 reads, which had an average length between 1,176 and 1,265 nt (Table S1). Full-length reads accounted for more than 93.12% of the total clean reads after filtering the rRNAs in each sample (Table S2). These reads had an N50 of 1295–1473 nt, and a maximum length of 15138–257481 nt. Using this dataset from the ONT sequencing platform, we identified 11,356 novel transcripts, 1,306 novel genes, and 375 lncRNAs.

Principal component analysis (PCA) and hierarchical cluster analysis based on the counts Per Million (CPM) of all transcripts in 12 samples revealed a large genotypic effect as well as a treatment effect (Figure S1A). To further investigate the degree of variability of the gene expression level distribution in each sample and to visually compare the overall gene expression levels of different samples, we constructed a boxplot to visualize the CPM distribution (Figure S1B). The overall expression of all samples remained at a relatively consistent level, and the expression levels of most genes were between 0 and 1.5.

### DET identification and functional analysis

**Figure 1:**
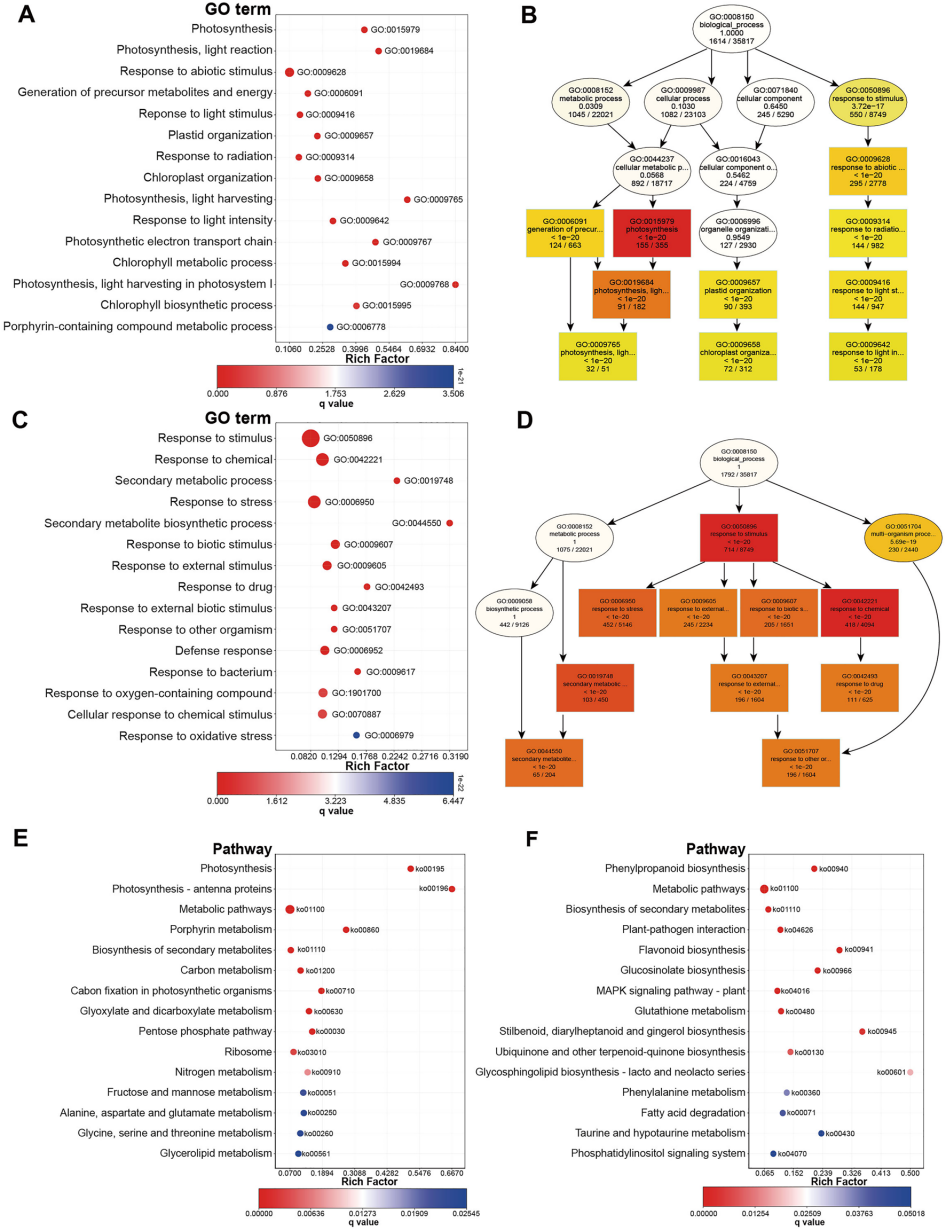
GO enrichment analyses and KEGG enrichment using DETs between the C group vs D group.

To assess transcriptomic changes under nitrogen starvation, DETs were identified with an at least a 1.5-fold difference in expression and an FDR less than 0.05 were identified for four comparisons: Col-0 + N (A) versus *atg5-1* + N (B), Col-0 + N versus Col-0-N (C), *atg5-1* + N versus *atg5-1*-N (D), and Col-0-N versus *atg5-1*-N. Interestingly, compared with nitrogen-rich conditions, WT plants exhibited substantial alterations in transcript expression after low-nitrogen treatment. In contrast, the mutation of *atg5-1* resulted in fewer changes in transcript expression under + /− N (Figure S2, Table S3). For the comparison between the C and D groups, we obtained a total of 5,797 DETs, of which 2,837 were upregulated and 2,960 were downregulated in group D (Table S3). To select the DETs that were specifically expressed in C versus D, we removed the DETs that were already present in A versus B. On this basis, a total of 5,545 DETs were found, of which 2,719 and 2,826 DETs were upregulated and downregulated in group D, respectively (Table S4). To analyze the functions of these DETs, GO enrichment analyses were performed. The results showed that these transcripts were associated with different functions in the biological processes category. Among the DETs under nitrogen starvation, the upregulated transcripts were mainly associated with response to stimuli, response to stress, and response to chemical processes (Fig. 1A,B). and the downregulated transcripts were mainly related to stimulus response, abiotic stimulus response, and photosynthesis (Fig. 1C,D). The above results not only show that autophagy is involved in the regulation of a wide range of stimuli, whether upregulated or downregulated, but also has a significant impact on photosynthesis.

In addition, to identify the metabolic pathways underlying nitrogen deficiency, a KEGG pathway enrichment analysis was performed. In addition to the secondary metabolic pathways and biosynthesis, the upregulated DETs in the WT were associated with phenylpropanoid biosynthesis (Fig. 1E). Carbon metabolism and photosynthesis were identified as being involved with downregulated transcripts (Fig. 1F).

**Figure 2:**
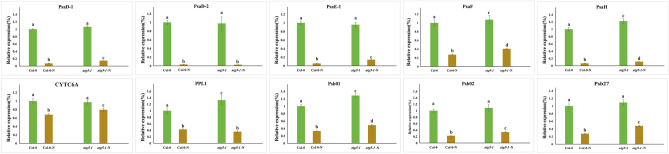
Expression analysis(+ /−N) of the 10 photosynthesis-related genes was performed via qRT-PCR. The error bars represent the standard errors of three replications, and the lowercase letter above the bar indicates a significant difference (α = 0.05, LSD) among the treatments.

Based on the KEGG and GO databases, we selected 10 genes associated with photosynthesis and their expression after nitrogen starvation for further study, which included photosynthetic electron transport chains I (PSI) and II (PSII), for further study. Similar to the results of RNA-seq, most of the photosynthesis genes measured (9/10) were expressed at higher levels in *atg5-1* than in Col-0 after -N treatment (Fig. 2). In addition to -N treatment, -C treatment is also a conventional means to induce autophagy. Therefore, we further investigated the expression levels of these photosynthesis genes before and after + /−C treatment in autophagy mutants. Unlike the -N treatment, only one PSI gene, PSAH, was more highly expressed in *atg5-1* than in Col-0 after -C treatment (Figure S3). However, after -C treatment, the expression of *CYTC6A*, *PPL1*, *PSBO1*, and *PSB27* was lower in *atg5-1* than in Col-0 (Figure S3).

### Determination of the photosynthetic index

**Figure 3:**
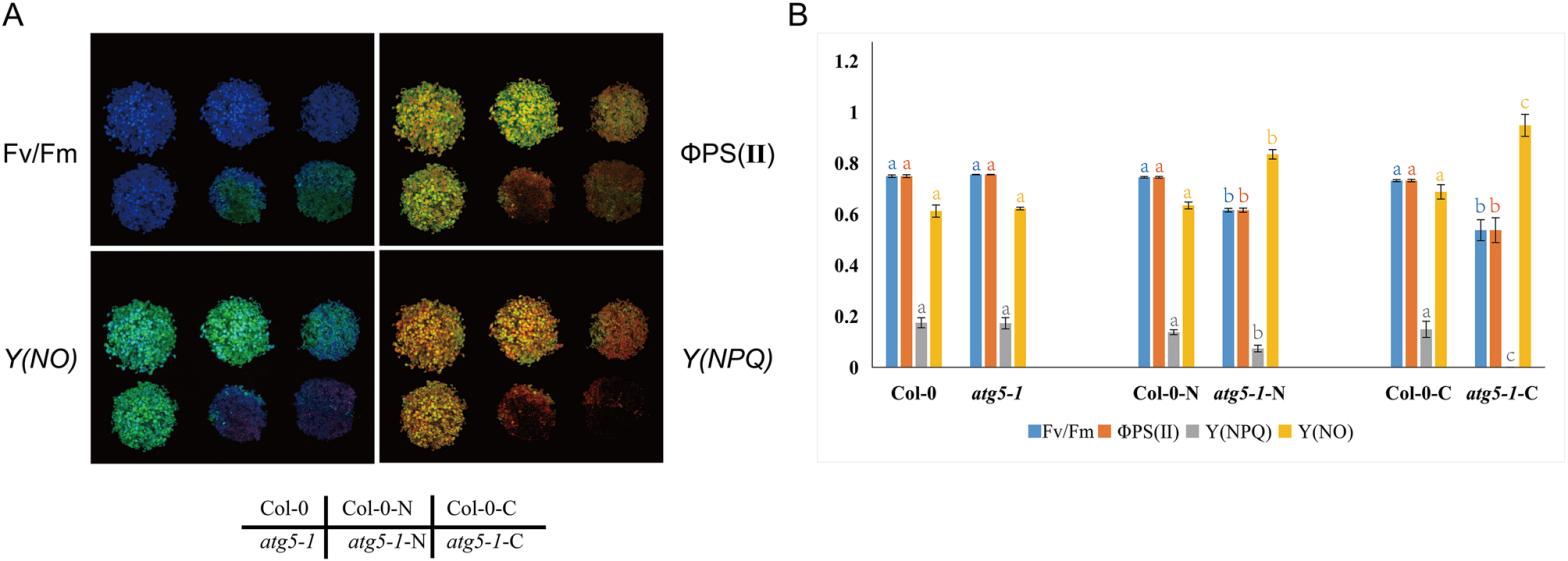
Photosynthesis index statistics of seedling. (**A**) Chlorophyll fluorescence images. (**B**) Photosynthetic indices under the condition the condition of nitrogen/ carbon starvation conditions at 56 μmol·m^−2^·s^−1^.

Given that the abundances of transcripts associated with photosynthesis were greater in the *atg5-1* background, assumed that autophagy may play an important role in the regulation of photosynthesis. To confirm this hypothesis, we used a chlorophyll fluorescence imaging system to analyze the photosynthetic indices of the abovementioned materials, under nitrogen/carbon starvation treatment, which was used to induce autophagy^45^. We measured the four main photosynthetic indices: Fv/Fm (PSII maximum photochemical quantum yield), ΦPSII (PSII efficiency), Y(NPQ)(the quantum yield of regulated energy dissipation (Y(NPQ)) and Y(NO)(the quantum yield of nonregulated energy dissipation (Y(NO)). Fv/Fm is the maximum quantum yield of PS II, reflecting the potential maximum photosynthetic capacity (photosynthetic efficiency) of a plant^46–48^. Compared with those of Col-0, the Fv/Fm, ΦPSII and Y*(NPQ)* of *atg5-1* decreased significantly after two days of nitrogen/carbon starvation. Conversely, the *Y(NO)* value decreased significantly (Fig. 3).

**Figure 4:**
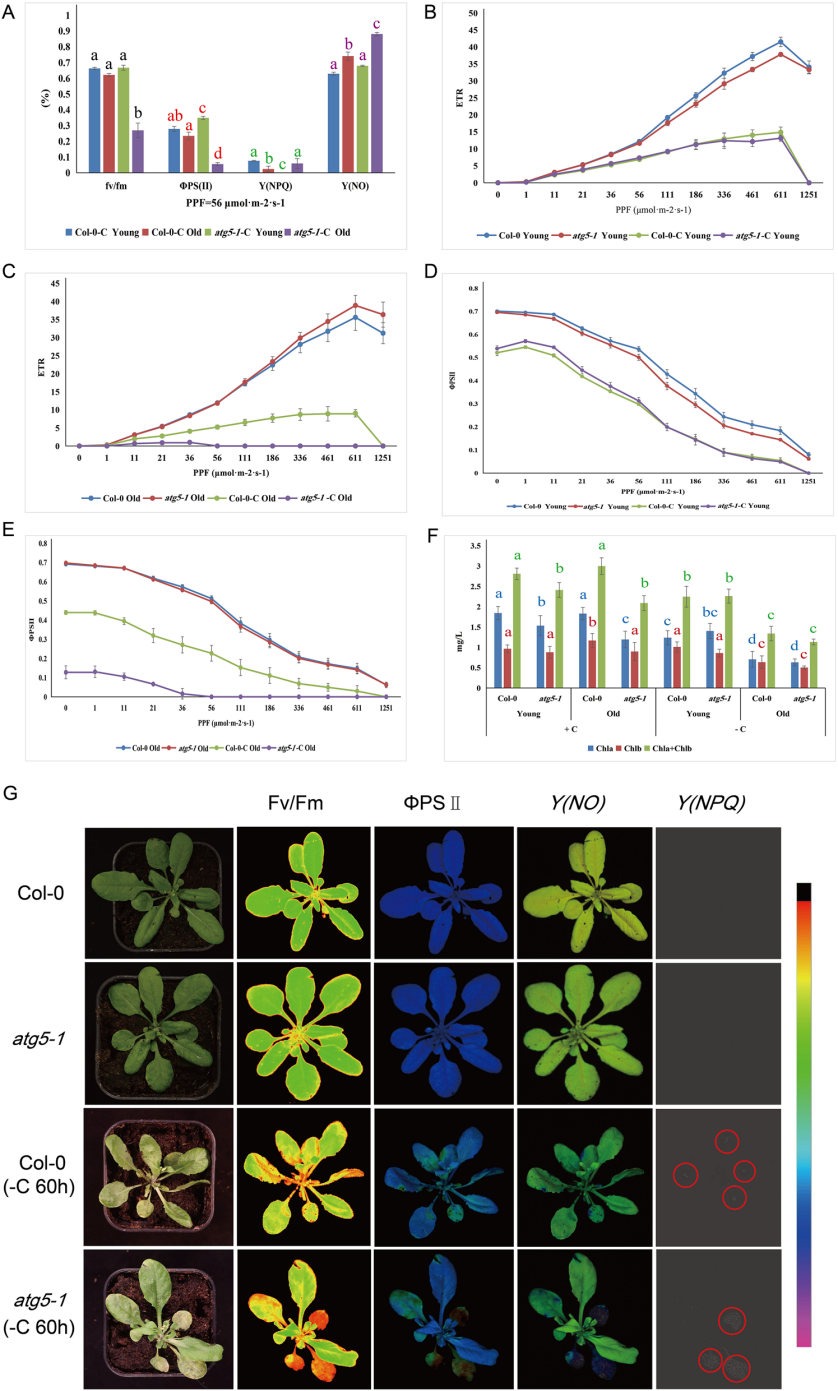
Photosynthesis index statistics of four-week-old seedlings. (**A**) Photosynthetic indices under the condition the condition of carbon starvation conditions at 56 μmol·m^−2^·s^−1^. (**B**) (Yong leaf), (**C**) (Old leaf), Photosynthetic electron transfer rate (ETR) under different photosynthetically active radiation (0 μmol·m^−2^·s^−1^–1251 μmol·m^−2^·s^−1^). PPF refers to the photosynthetic photon flux. (**D**) (yong leaf), (**E**) (old leaf), and ΦPSII under different photosynthetically active radiation treatments. (**F**) Determination of chlorophyll content. G The chlorophyll fluorescence images. The red circle indicates the site where NPQ occurs occurred.

However, as shown in Fig. 3A, even the same line exhibited different phenotypes under the same treatment conditions. For example, the image of *Y(NPQ)* shows that different leaves on the same plant will also show different colors (green, yellow, and red). Therefore, to further validate this result, we measured the photosynthetic indices of four-week-old plants to determine whether there was a difference between new and old leaves. As shown in Fig. 4G and Figure S4, no significant difference was detected in the photosynthetic indices including Fv/Fm between Col-0 and *atg5-1* in both young and old leaves under normal conditions.Under fixed-carbon starvation, a sharp increase in *Y(NO)* and a decrease in Fv/Fm and ΦPSII were observed in old leaves of the *atg5-1* mutant compared to young leaves of Col-0 and *atg5-1* and old leaves of Col-0, respectively (Fig. 4A, G, S4). The results showed that Fv/Fm was approximately 0.7 in the young leaves of the WT and *atg5-1* plants, and + /− C led to a significant difference between them. In contrast, the Fv/Fm decreased rapidly to approximately 0.27 in the old leaves of *atg5-1* (Fig. 4A, G, S4). Both the quantum yield of regulated energy dissipation (*Y*(*NPQ*)) and the quantum yield of nonregulated energy dissipation (*Y*(*NO*)) are indicators of energy dissipation during photosynthesis. In the normal growth environment without nutritional stress, the *Y*(*NPQ*) values of the WT and *atg5-1* plants were both 0. However, under the carbon conditions, the *Y*(*NPQ*) value of young WT leaves was significantly greater than that of young *atg5-1* leaves. Surprisingly, *Y*(*NPQ*) showed the opposite trend in the old leaves of both plants (Fig. 4A, G, S4). Similar to *Y*(*NPQ*), WT and *atg5-1* showed similar *Y*(*NO*), values of approximately 6.5 to 6.9, under nonstress conditions. (Fig. 4A, G, S4). After carbon starvation, the *Y*(*NO*) values of the young leaves of the WT and mutant plants did not change significantly but increased significantly in old leaves, the value for *atg5-1* increased to approximately 0.88, which was significantly greater than the value of 0.74 was observed for the WT. In addition,the results showed that the ΦPSII values of the WT and *atg5-1* in the plants without stress treatment were stable between 0.51 and 0.52 in the old and young leaves of *atg5-1* and decreased to 0.06 and 0.35, respectively (Fig. 4A, G, S4). Furthermore, we compared the differences between the relative electron transport rate (*rETR* (II)) and ΦPSII under gradually increasing light intensity from 0 to 1251 μmol·m^−2^·s^−1^. Apparently, the young leaves of the WT and *atg5-1* plants exhibited similar patterns of *rETR* (II) and Φ PSII (Fig. 4B, D). In contrast, in old leaves under fixed-carbon starvation, *atg5-1 rETR* (II) and ΦPSII were lower in *atg5-1* than in the WT (Fig. 4C, E).

To determine whether the changes in the photosynthetic indices of old *atg5-1* leaves were caused by the degradation of chlorophyll, we examined the chlorophyll contents of plant leaves. Under normal growth conditions, the chlorophyll content in the old leaves of *atg5-1* was slightly lower than that in old WT leaves, but there was no significant difference in the chlorophyll content in the new leaves (Fig. 4F). A similar trend was observed after nutritional stress (Fig. 4F).

### Characterization of AS events

**Figure 5:**
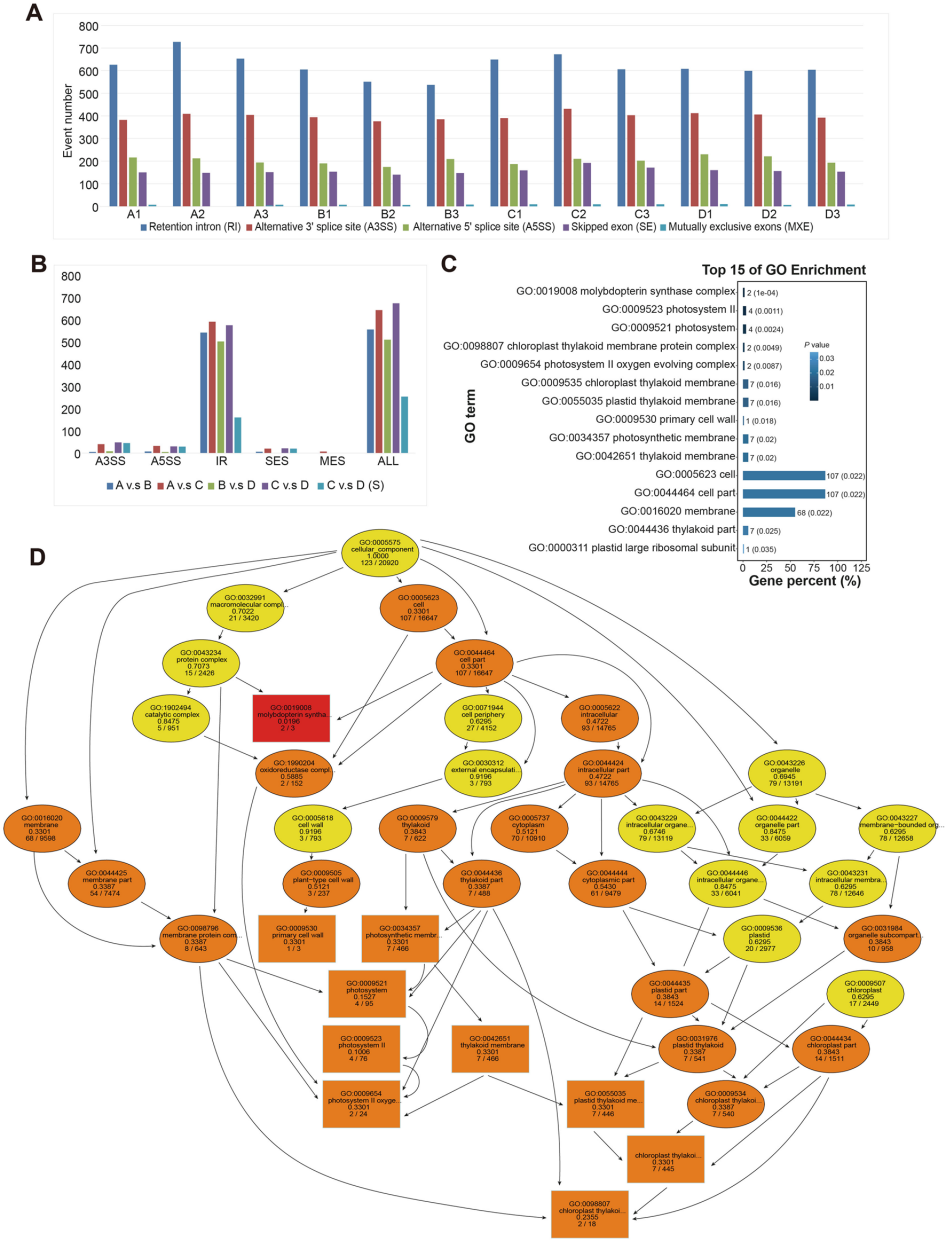
Identification of alternative splicing (AS) events and functional analysis. (**A**) Statistics of the number of AS events in each sample. (**B**) Comparison of AS events between different groups C vs D(S) indicated that the AS event occurred only in the C vs D group. (**C**) Filtered differential alternative splicing events between groups. A3SS, A5SS, IR, ES, MES, and ALL indicate alternative 3’ splice site, alternative 5’ splice sites, intron retention, exon skipping, mutually exclusive exons, and total AS events, respectively. (**D**) Many genes involved in AS events are involved in photosynthesis.

Increasing evidence indicates that AS plays a crucial role in plant development and stress response. Five categories of AS events were identified in this study, the most common event was intron retention (41.76 to 48.47%), and the least abundant event was mutually exclusive exons (0.27% to 0.7%) (Fig. 5A). Under high nitrogen levels, the number of AS events, particularly intron retention events, was higher in the WT than in *atg5-1* (Fig. 5A). In contrast, there was no significant difference in the total number of AS events between the WT and the mutant *atg5-1* under low nitrogen levels, but the proportion of intron retention events was greater in the WT (Fig. 5A). Furthermore, we comprehensively compared AS events among the four groups. The overall trend was more consistent with the results for DETs; that is, the WT had more AS events (A vs. C, 643) under nitrogen starvation, while there were relatively few AS events in *atg5-1* (B vs. D, 510) (Fig. 5C, D). Regardless of which groups were compared, intron retention was the most common AS mode.

To select the ASs that changed specifically between C vs D, we removed the ASs that were already present between A vs. B. We finally screened 254 ASs that changed specifically in the C vs D group from the 674 AS events (Table S5, Fig. 5B). Next, we performed GO enrichment analyses on the 254 genes with AS events. Surprisingly, many transcripts (78) of these AS events were also linked to photosynthesis. For example, several genes were associated with the photosynthetic system, chloroplast thylakoid membrane, etc. (Fig. 5C, D); Table S6).

### Characterization of lncRNAs

LncRNAs were identified by CNCI, CPAT, CPC, and Pfam analyses, and a total of 375 lncRNAs were detected using all four methods (Figure S5). LncRNAs were classified and mapped according to their position on the reference genome annotation information (gff). All lncRNAs were classified into three categories with 233 lncRNAs, 54 antisense lncRNAs, 0 intronic lncRNAs, and 88 sense lncRNAs (Figure S5). Among the 375 lncRNAs, 43 were differentially expressed in the C vs. D group, and 20/23 were up-/downregulated in Col-0 under nitrogen starvation (Table S7). Furthermore, 6 lncRNAs were predicted to target autophagy-related genes (Table S7), possibly through a negative feedback mechanism role during autophagy occurrence.

## Discussion

Autophagy plays a key role in plant resistance to nutrient stress. Nitrogen is a necessary macroelement for plants and nitrogen deficiency leads to significant changes in the transcriptome of plants. To accurately characterize gene expression patterns and molecular traits under nitrogen starvation, some studies have assessed transcriptomic changes using the Illumina sequencing platform^15,17,49^ Because mRNA transcripts are the direct template for translation, identifying changes that affect the abundance of autophagy transcripts under nitrogen starvation conditions is an important task. In this study, we performed full-length RNA sequencing of Arabidopsis Col-0 and *atg5-1* plants treated with or without nitrogen. A total of 39,033 transcripts were detected, including 11,356 novel transcripts. We identified 2,719 upregulated DETs and 2,826 DETs in *atg5-1* under nitrogen starvation conditions by comparative transcriptomic analysis (Table S4).

By analyzing DETs, several potential molecular regulatory networks were revealed. We found that there were only 412 DETs from the WT and the autophagic mutant *atg5-1* when sufficient nitrogen was present (Table S3). Accordingly, there is a were many DETs (5797) under nitrogen starvation (Table S3). This result may suggest that autophagy is necessary to maintain plant homeostasis under nitrogen starvation, while it appears to have little effect on plant growth, at least at the seedling stage under sufficient nitrogen. In addition, we found that there was no significant difference in the expression of most transcripts of the *atg5-1* transcripts between the +/−N conditions (Table S3). This further suggested that autophagy is essential for the regulation of plant nutritional stress.

To further analyze the function of up- and downregulated DETs between the C group (*atg5-1* + N versus *atg5-1*-N) and D group (Col-0-N versus *atg5-1*-N), we performed the GO and KEGG enrichment analyses. From the GO results, we found that many transcripts in both up- and downregulated DETs were involved in stimuli (Fig. 1). Autophagy plays two roles in regulating plant abiotic stress: it can upregulate the expression of some stress response genes and also downregulate the expression of other stress genes. GO and KEGG analyses, revealed that the photosynthesis-related transcripts of the autophagy mutant *atg5-1* had many of that were significantly upregulated under nitrogen starvation (Fig. 1). This indicates that plants weaken photosynthesis through autophagy to cope with nitrogen starvation and even abiotic stress.

Compared to previous RNA-seq results, we found that different sample periods and processing times affected the final sequencing results. For example, Masclaux-Daubresse et al. sequenced seedlings subjected to low-nitrogen treatment for 30 and 60 days, while we treated them with 5-day-old seedlings for 2 days. The enrichment of genes associated with photosynthesis was not mentioned in their study. Shi et al. reported that 16 h of low-nitrogen treatment also enriched photosynthesis-related genes. This may be because the low nitrogen stress in the seedling stage preferentially affects the photosynthesis-related genes, while some metabolites or senescence-related genes are more concentrated in larger plants.

The RNA-seq results were further validated by qRT-PCR (Fig. 2). In the present study, we were not been able to determine the cause of the increase in the expression of photosynthesis-related genes in a low-nitrogen environment, possibly because too much photosynthesis can cause damage to plants. Conversely, the expression of most PSII-related genes in the mutants decreased significantly after -C treatment, suggesting that autophagy may affect photosynthesis by affecting the expression of PSII-related genes. Autophagy may be involved in PSII photoprotection and repair under stress/dark conditions and ultimately affects photosynthetic efficiency^50,51^. The difference in the expression of photosynthesis genes in *atg5-1* under low nitrogen and low carbon conditions indicates the complexity of autophagy in regulating photosynthesis, which may involve the influence of autophagy on the plant C/N balance. More experimental evidence at the protein level should be obtained in the future to validate the results at these transcriptional levels. PSII-related genes, such as *PsbO1*, *PsbO2* and *Psb27* identified in this study, may be degraded by autophagy as potential target genes. Among them, PsbO encodes an extrinsic subunit of photosystem II, which includes *PsbO1* and *PsbO2*, and has been proposed to play a central role in catalyzing the stabilization of manganese clusters^52,53^. PsbO1 mutations cause PSII to fail on both the donor and acceptor sides, and the PSII center is highly sensitive to photodamage, however, PsbO2 mutations do not appear to affect the main body function of PSII, and PsbO2 may only affect the effective repair of PSII complex under high light^53^. Studies have shown that CV (for chloroplast vesiculation) targets chloroplast degradation by interacting with PsbO1 under the activation of aging or abiotic stress. Our study suggested that the degradation of PsbO1 is regulated by autophagy^54^. Similar to Psbo1, Psb27 is not necessary for aerobic photosynthesis and PSII formation. In contrast, Psb27 is involved in the recovery process of PSII as an luminal protein^55^. Another study suggested that Psb27 may also be involved in the photoadaptation of *Arabidopsis thaliana* at low temperatures, which is the result of natural variation^56^. In conclusion, our results suggest that autophagy is involved in photodamage repair in plants, and future studies can focus on the degradation of these genes, such as *PsbO1*, *PsbO1*, and *Psb27*, as well as other genes associated with photo repair that have not yet been studied.

Based on the results of GO and KEGG analyses, many photosynthesis-realted genes were downregulated in the WT plants compared to those in the *atg5-1* plants, which may indicate that autophagy has a direct or indirect relationship with regulating the transcription of photosynthesis-related genes. To date, there are few reports on autophagy and photosynthesis in plants. A recent study showed that plants can reduce high-intensity light damage by accumulating ROS through pexophagy and microautophagy^57^. This result indicates a relationship between autophagy and high-intensity stress, which also suggests that autophagy is related to photosynthesis. Using chlorophyll fluorescence imaging, we further confirmed that autophagy can regulate photosynthesis in plants via unknown mechanism. Fv/Fm and ΦPSII represent the maximum and real quantum efficiency of photosystem II, respectively. The higher the value is, the stronger the photosynthesis. It is clear that at the seedling stage, Col-0 does not appear to be very sensitive to short-term (within 2 days) nitrogen/carbon starvation. There was no significant difference in photosynthetic efficiency or photosynthetic damage between the seedlings before treatment. In our experience, Col-0 remains green at approximately 10 d/12 d of nitrogen/carbon starvation^9^. This may be because there are still some nutrients remaining in the seeds that have not been exhausted. Conversely, *atg5-1* appears to be very sensitive to starvation. Even though 2 days of starvation treatment did not cause the plants to turn yellow significantly, the decrease in photosynthetic efficiency and increase in photosynthetic damage was very obvious. Next, we obtained new results by measuring photosynthesis in different leaves of four-week-old plants. We found that after starvation treatment, the Fv/Fm and ΦPSII decreased significantly in the old leaves of the autophagic mutant *atg5-1*, reflecting a decrease in photosynthesis (Fig. 4A). The same trend was observed under the different lighting conditions (Fig. 4C). *Y*(*NPQ*) and* Y*(*NO*) represent the regulatory and nonregulatory energy dissipation of PSII, respectively. A high *Y*(*NPQ*) indicates that plants have a strong photoprotection mechanism, such as heat dissipation and other forms of dissipation of excess light energy^58^. In normally growing plants (WT and *atg5-1*), we found that the *Y*(*NPQ*) values were all 0, which may be due to the lack of excess light energy such that the photoprotective mechanism was not activated. However, in the young leaves after nitrogen starvation, the WT *Y*(*NPQ*) value increased to 0.08 ± 0.008, while that for *atg5-1* was still 0 (Fig. 3A). This may be due to the weakening of photosynthesis caused by stress, suggesting that the WT plants were better protected under strong light. A high *Y*(*NO*) value indicates that the photochemical PSII reaction and protective regulatory mechanism play no role and that excessive excitation energy damages PSII Ref.^58^. Apparently, the *Y*(*NO*) value of the old leaves of *atg5-1* after nitrogen starvation reached 0.88 ± 0.011, while that of the WT leaves was 0.741 ± 0.026, further indicating that the photoprotective ability of the autophagic mutant was weaker (Fig. 4A). Chlorophyll levels can also be an indicator of the photosynthetic capacity of plants. We found no significant change in the chlorophyll content in young WT leaves and *atg5-1* after treatment. A decrease in chlorophyll content in old *atg5-1* old leaves was observed during normal growth, which may indicate that autophagy does not regulate photosynthesis by controlling chlorophyll levels in plants under nutrient stress. Curiously, there was no significant difference in chlorophyll content between the WT and mutant plants in either old or new leaves after carbon stress (Fig. 4F), suggesting that autophagy may not be involved in chlorophyll degradation in the light-avoidance reaction. However, studies have shown that the selective autophagy receptor NBR1 degrades the translocon at the outer envelope embrane of chloroplasts (TOC) under some abiotic stresses such as UV-B irradiation and heat stress^59^. This suggests that autophagy, as one of the pathways of chloroplast degradation, only occurs under specific conditions, and requires further research. Notably, all changes in photosynthetic indices occurred in relatively old leaves, while there were only minor changes in new and young leaves. This suggests that plants respond to stress by preferentially transporting nutrients to young leaves to ensure plant survival. When the autophagy pathway is blocked, plants cannot gain energy from the breakdown of intracellular substances, leading to senescence and death of old leaves. Chloroplasts are the most important sites for photosynthesis. Two new studies have shown that autophagy is extensively involved in chloroplast degradation under abiotic stress, which may be one of the main reasons for the decline in plant photosynthetic capacity^59,60^.

In eukaryotes, AS is a posttranscriptional regulatiory mechanism for the production of new transcripts. In this study, we also examined two events in the WT and *atg5-1* plants under + /−N conditions. The overall result was similar to the trend for DETs observed previously, that is, the WT showed more genes becoming subject to AS under nitrogen starvation, while *atg5-1* was relatively insensitive. Alternatively, autophagy can specifically degrade or affect the configuration of these photosynthesis genes. GO enrichment analyses revealed that numerous genes related to photosynthesis had AS events (Fig. 4C; Table S6). This finding suggested that autophagy plays an important role in the breakdown of chloroplasts and photosynthetic system II. Interestingly, a gene called CV (AT2G25625), which is associated with the photosynthetic membrane was previously reported to regulate plant senescence independently of the autophagic pathway^54^.

In addition, we identified 20 and 23 lncRNAs whose expression increased and decreased, respectively, in the WT plants under nitrogen deficiency stress. Among these lncRNAs, 6 have the the potential to regulate autophagy (Table S7). The target genes of these lncRNAs include autophagy-related genes, particularly *ATG8*, which may be involved in autophagy. Notably, four *ATG8* genes (*ATG8b*, *ATG8d*, *ATG8g*, and *ATG8h*) were separately regulated by different lncRNAs, suggesting that *atg8* genes may exhibit different expression patterns during stress generation (Table S7). Among the 4 lncRNAs that regulate *atg8*, three were upregulated and one was downregulated, suggesting that these different lncRNAs play a role in promoting/inhibiting autophagy genes. ATG8 is a ubiquitin-fold protein that becomes attached to phosphatidylethanolamine and acts as a docking platform for autophagic receptors and adaptors^1^. ATG8 is the core autophagic protein that determines whether autophagy can degrade its target. In addition, the lipidized ATG8 protein completes the process of selective autophagy by binding to a group of autophagy receptors that have an affinity for specific cargo^61–63^. There are 9 ATG8 proteins in Arabidopsis^64^, and the current methods for monitoring autophagosomes generally involve GFP-ATG8a or GFP8-ATG8e transgenic lines from the Richard D. Vierstra and Liwen Jiang laboratories, respectively^65,66^.However, whether there are differences in the function of selective autophagy between different ATG8 proteins, whether they regulate the degradation of different proteins/organelles, or whether they have functional redundancy still needs to be further studied. In addition, the upstream regulators of the ATG8 protein have rarely been reported. In our study, we identified four lncRNAs that can regulate the functions of different ATG8 proteins, and the next step was to knock out these lncRNAs by gene editing or RNAi to study the functions of different ATG8 proteins.

## Materials and methods

### Plant materials and treatment

The seeds of the *Arabidopsis* ecotypes Col-0 and *atg5-1*^65^ were surface sterilized with 1% sodium hypochlorite after 3 days of stratification at 4 °C. The sterilized seeds were grown in a triangular flask with liquid MS culture media for 5 days under a long- day photoperiod (16 h light/8 h dark) with a continuous temperature of 22 °C and shaking at 100 rpm. Plants that were well transferred to MS or nitrogen-depleted liquid media for an additional 2 days, were used as the control and treatment groups, respectively. The whole seedling tissue was used for subsequent sequencing and experiments.

### RNA-seq library construction and Nanopore sequencing

The experimental procedure was performed according to the standard protocol provided by ONT. Briefly, RNA from Arabidopsis seedlings was extracted with a plant RNA isolation kit (RC401-01, Vazyme). A cDNA PCR barcoding kit (SQK-PCS109 with SQK-PBK004, ONT) was used for sample cDNA generation, barcoding and sequencing. A total of 12 samples, including 3 biological replicates of each treatment or control, were sequenced. For each sample, 2 ng of polyA + RNA was used for reverse transcription and strand switching, and 5 µL of reverse transcribed RNA was used to select for full-length transcripts. The cDNA product was amplified for 14 cycles with LongAmp® Taq DNA Polymerase (NEB). Then, adapters were added to the cDNA samples by T4 DNA ligase (NEB). The final cDNA libraries were added to FLO-MIN109 flow cells, and sequenced on the PromethION platform from Biomarker Technology Company (Beijing, China).

### Raw data processing and genome mapping

The low-quality reads (Q-score < 6, length < 200 bp) were filtered and ribosomal RNAs were discarded after mapping to the rRNA database (https://www.arb-silva.de). After trimming the adapter primers, the full-length nonchimeric transcripts were mapped to the Arabidopsis TAIR10 reference genome by minmap2 (https://github.com/lh3/minimap2) Ref.^67^ and further polished to obtain consensus sequences by pinfish (https://github.com/nanoporetech/pinfish). Principal component analysis (PCA) was performed using BMKCloud (www.biocloud.net).

### Differential gene/transcript expression analysis

The counts per million (CPM) calculation method was used to standardize the number of reads versus the genome as a data indicator to measure the expression level of transcripts. The nonredundant full-length sequences were compared to the reference transcriptome, and the quantitative results of all transcripts transcribed from the gene were counted as the quantitative results of the gene, which were also calculated by the CPM method. For the quantitative results of transcripts and genes, DESeq2 was used for difference analysis and the p-value was corrected by the Benjamini–Hochberg method^68^ to obtain the false discovery rate (FDR). Transcripts with a fold change (FC) ≥ 1.5 and FDR < 0.05 were selected for further analysis.

### Alternative splicing analysis

Transcripts were validated against known reference transcript annotations using gffcompare^69^. AS events were detected by the AStalavista tool^70^. Different types of AS events, including alternative 3' and an alternative 5' splice sites, as well as exon skipping, intron retention, and mutually exclusive exons, were identified.

### Identification of long noncoding RNAs

We identified CDSs of polished nonredundant isoforms using TransDecoder software v3.0.0 (https://github.com/TransDecoder/TransDecoder) Ref.^71^. Prediction of lncRNAs was performed using four methods, the Coding Potential Calculator (CPC, v0.9-r2; https://github.com/biocoder/cpc) Ref.^72^, the Coding-Non-Coding Index (CNCI, v2; https://github.com/bioshare/CNCI) Ref.^73^, the Coding Potential Assessment Tool (CPAT, v1.2.2; https://rna-cpat.sourceforge.net/) Ref.^74^, and Pfam (v1.3; https://github.com/fpozoc/hp-pfamscan). These four methods were used in combination to screen out the noncoding RNA sequences of noncoding proteins from the predicted RNA sequences of coding proteins. The screening criteria for lncRNA candidates were based on transcript length and exon number (greater than 200 bp in length with more than two exons). LncRNAs were then classified as intergenic lncRNA (also called lincRNAs), antisense lncRNAs, sense lncRNAs, or intronic lncRNAs. Target genes regulated by the identified lncRNAs were also predicted using the lncTar (v1.0) software (http://www.cuilab.cn/lnctar) Ref.^75^.

### Functional annotation and enrichment analysis

Gene/transcript functions were annotated based on the GO^76^ and KEGG^77^ databases. We used the GOseq R package (v3.0, https://github.com/xmao/kobas) Ref.^78^ and KOBAS (v3.0, https://github.com/xmao/kobas) software^79^ to perform GO and KEGG enrichment analyses on DEGs, respectively.

### Measurement of the photosynthetic index and chlorophyll content

For seeding: seeds stored at 4 °C for three days were transferred to a six-well plate, added to MS liquid media, and then placed on a shaker for growth (16 h light/8 h dark, 22 °C) for one week. After the MS liquid media was fully aspirated with a pipette, fresh MS liquid media or MS-N/-C media.was added. The samples were incubated on a shaker for two days. Note: The -C treatment also requires protection from light. For four-week-old Arabidopsis plants, the photosynthetic index and chlorophyll content were measured. Chlorophyll fluorescence images were obtained using an IMAGING-PAM system (WALZ, Germany). The maximum quantum efficiency of photosystem II (Fv/Fm) was measured after 20 min dark of adaptation with a saturating light pulse and with a photosynthetic photon flux density (PFD) of 6,000 µmol m^−2^ s^−1^ for 1 s. Then, the plants were exposed to an actinic PFD of 56 µmol m^−2^ s^−1^. Saturation flashes were applied 10 times every 20 s, and the effective quantum efficiency of PSII (ΦPSII) after 224 s of light adaptation was used for data analysis. The photosynthetic electric transport rate (ETR) was determined by continuous irradiation with gradually increasing light intensity (0–1,251 µmol m^−2^ s^−1^) in 12 steps (20 s for each stage). We applied three replicates for each sample.

For determination of chlorophyll content, 0.1 g of freshly-harvested leaves was put into 5 ml of anhydrous ethanol, and stored in a dark incubator at 4 °C for 48 h to extract chlorophyll (mg/L). Chla = 13.95*A665-6.88*A649; Chlb = 24.96 × A649–7.32 × A665. There were 3 replicates per sample.

### RNA Isolation and qRT-PCR

Seven-day-old plants were treated with -N (4 d) or -C (2 d). The samples were quickly frozen in liquid nitrogen, after which RNA was extracted. The qRT-PCR primers used for the 10 genes involved in photosynthesis are are listed in Table S8A rapid RNA isolation kit (Vazyme, China) was used to extract total RNA from the samples. Total RNA was used for complementary cDNA synthesis using SuperScript II QRT SuperMix (Vazyme. China) in accordance with the manufacturer’s instructions. qRT-PCR analysis was performed on a Bio-Rad CFX96 instrument using 2 × ChamQ universal SYBR qPCR Master Mix (Vazyme. China). The PCR reaction conditions were as follows: 95 ℃ for 30 s, followed by 40 cycles of 95 ℃ for 5 s, and 60 ℃ for 30 s. The 2^−ΔΔCT^ methods was used to determine the relative expression levels of genes. The experiments were repeated three times.

### Statistics

For the chlorophyll content measurement, SPSS (version.20, United States) software was used for one-way analysis of variance (ANOVA). The means of the data were analyzed using Duncan’s test for statistical significance (P values ≤ 0.05). For the comparison of the number of ASs, the t-test was used to detect whether there was a significant difference in the number of AS between groups. The photosynthetic indexes measured at 56 μmol·m^-2^·s^-1^ are presented as the mean values ± standard errors (± SEs).

### Supplementary Information

Supplementary Figures.

Supplementary Table S1.

Supplementary Table S2.

Supplementary Table S3.

Supplementary Table S4.

Supplementary Table S5.

Supplementary Table S6.

Supplementary Table S7.

Supplementary Table S8.

## Data Availability

The raw sequencing data have been deposited in the National Genomics Data Center (NGDC), Beijing Institute of Genomics, Chinese Academy of Sciences/China National Center for Bioinformation (https://ngdc.cncb.ac.cn/), under the accession number PRJCA018751 (https://ngdc.cncb.ac.cn/gsa/s/essO5v6w).
