# Supplementary material for: Full-length transcriptome sequencing of Arabidopsis plants provided new insights into the autophagic regulation of photosynthesis
Source: Sci Rep. 2024 Jun 25;14:14588. doi: 10.1038/s41598-024-65555-7 (PMC11199623; doi:10.1038/s41598-024-65555-7)
Supplement: Supplementary file 1 — Supplementary Figures. [file 41598_2024_65555_MOESM1_ESM.pdf]

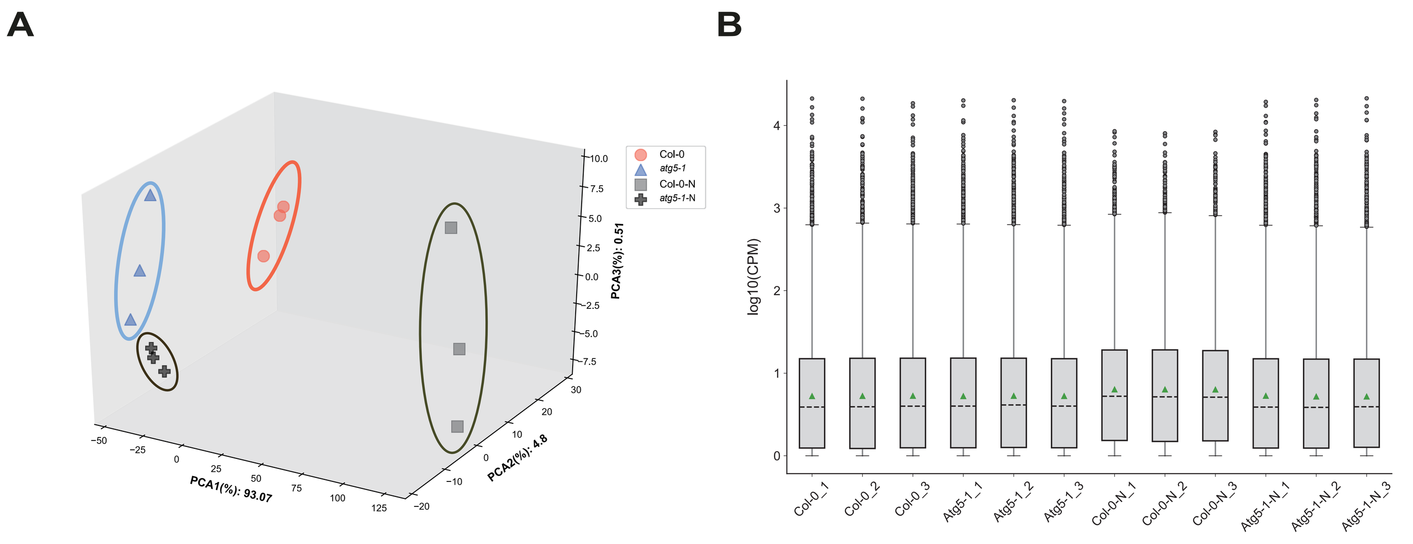

Figure S1 Overview of sequencing quality. A, Principal component analysis (PCA); B, CPM boxplot of each sa
Volcano plot of differentially expressed genes in each group.

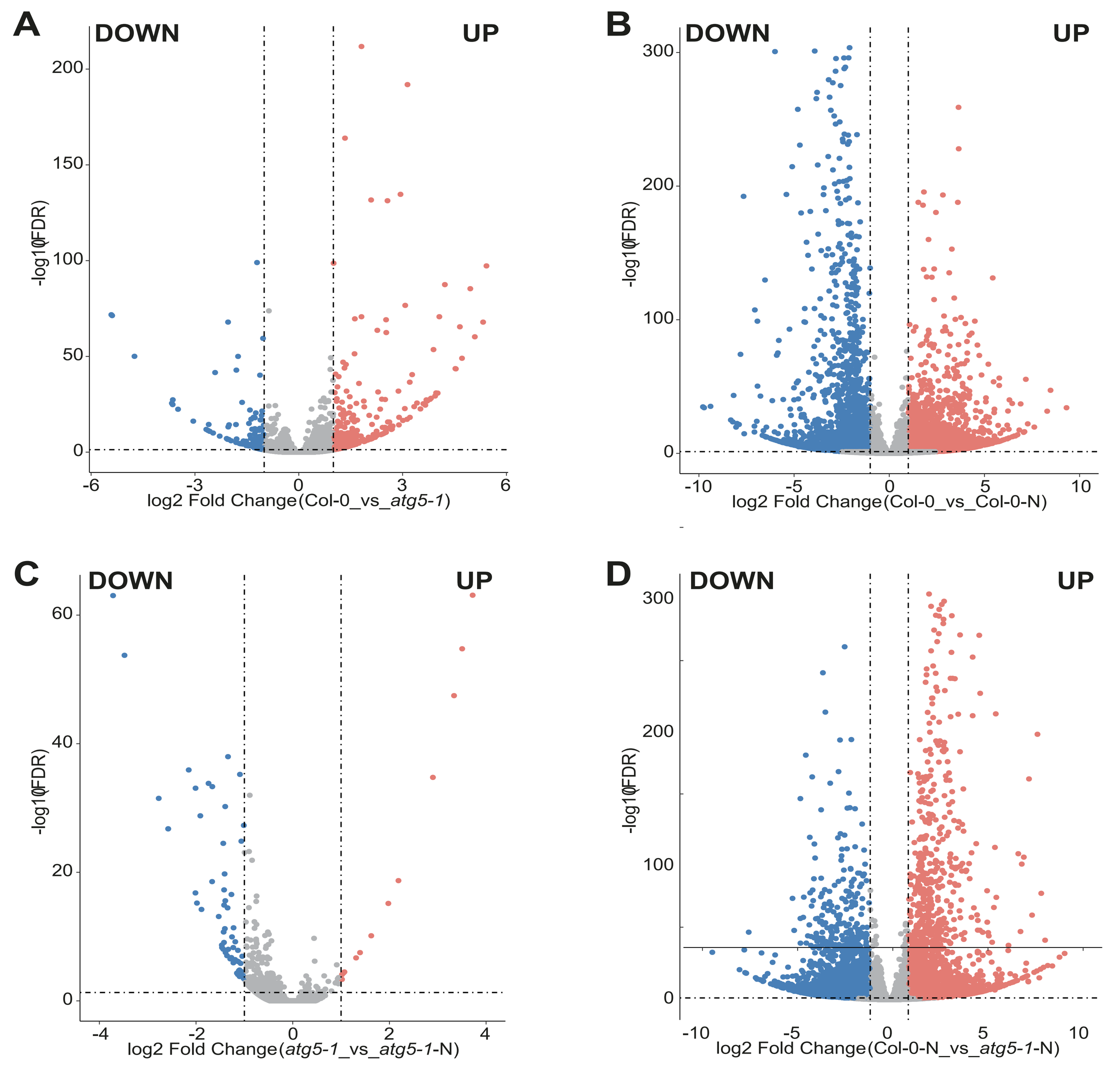

Figure S2 Volcano plot of differentially expressed genes in each group.

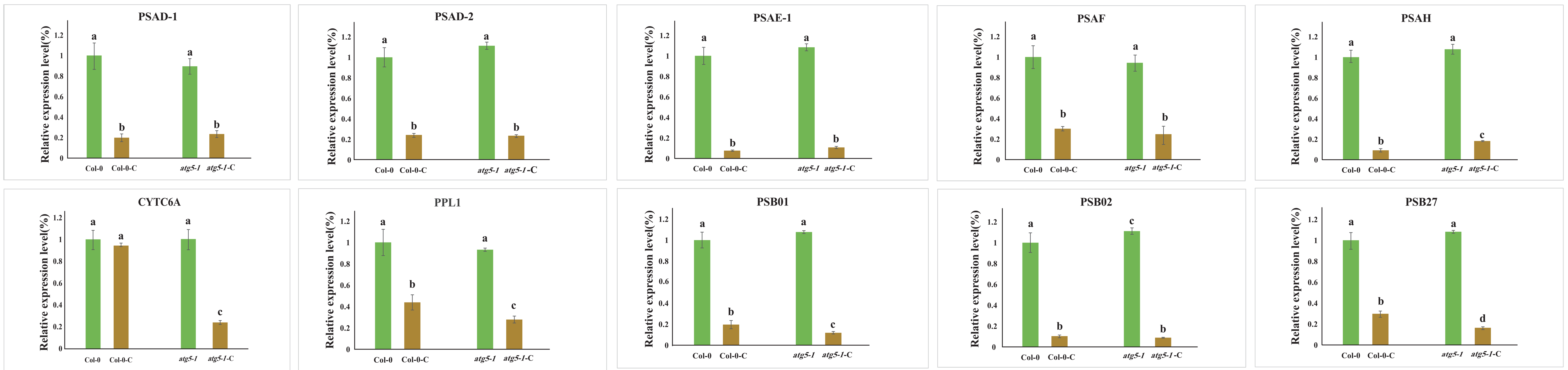

Figure S3 Expression analysis(+/-C) of the 10 photosynthesis-related genes was performed using qRT-PCR. Error bars represent the stand errors with three replications and the lowercase letter above the bar indicates a significant difference (α=0.05, LSD) among the treatments.

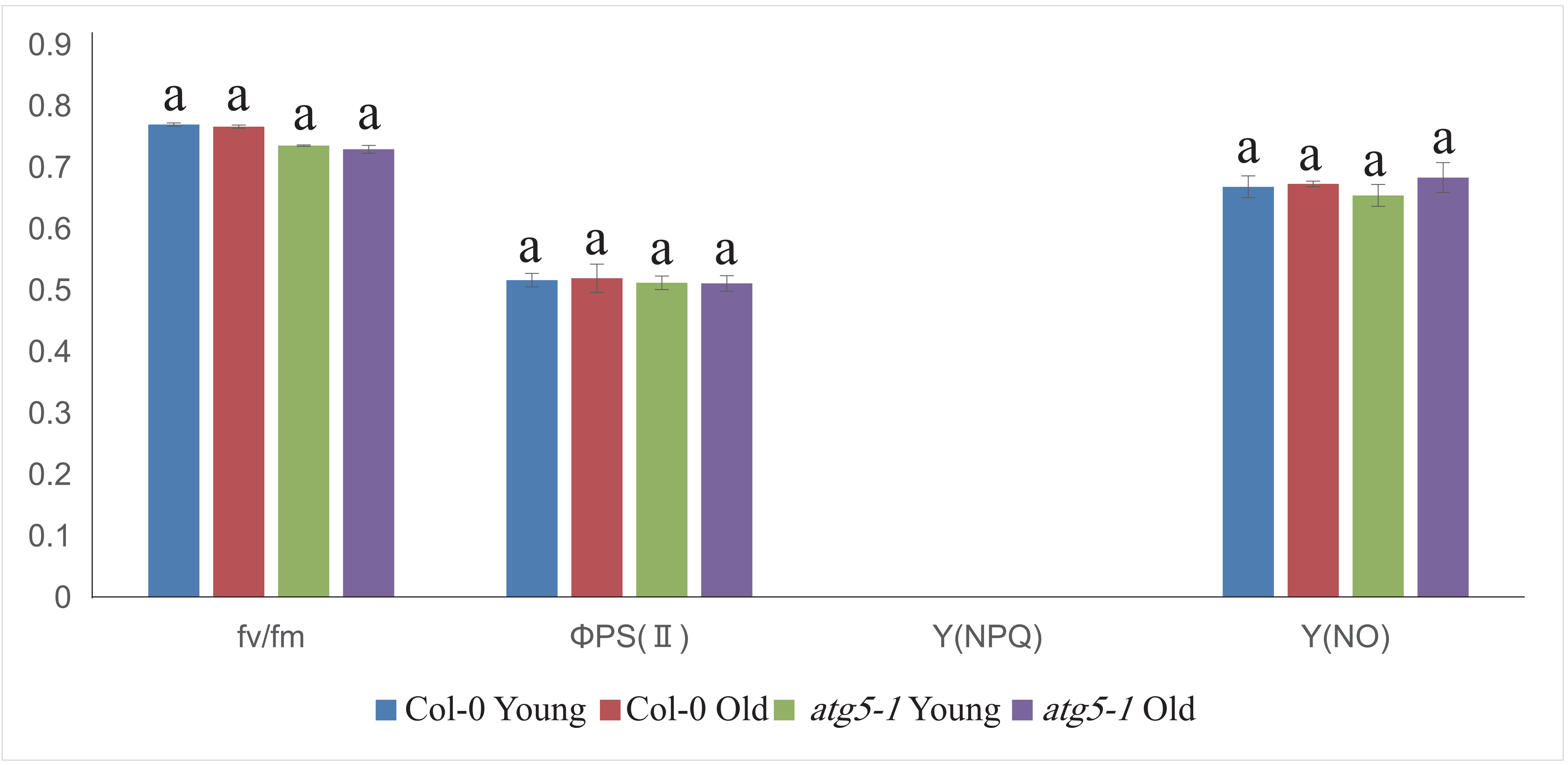

Figure S4 Photosynthetic index statistics under normal conditions. Photosynthetic index at 56 μmol·m-2·s-1.

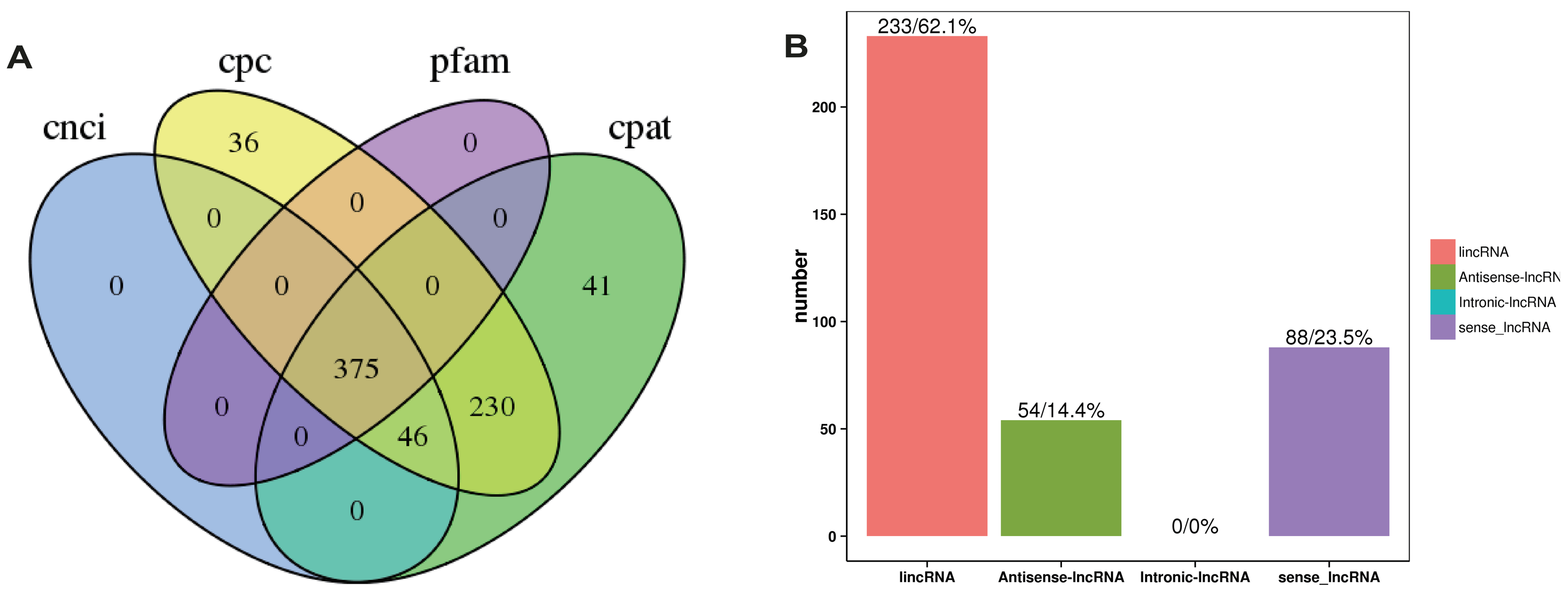

Figure S5 Identification and characterization of long non-coding RNAs (lncRNAs). A, A Venn diagram of the lncRNAs identified by four different methods. B, Statistics of the location of the lncRNAs.
